# Reactive arthritis induced by active extra-articular tuberculosis: A case report

**DOI:** 10.1097/MD.0000000000018008

**Published:** 2019-12-10

**Authors:** Yushiro Endo, Shin-ya Kawashiri, Tomohiro Koga, Momoko Okamoto, Sosuke Tsuji, Ayuko Takatani, Toshimasa Shimizu, Remi Sumiyoshi, Takashi Igawa, Naoki Iwamoto, Kunihiro Ichinose, Mami Tamai, Hideki Nakamura, Tomoki Origuchi, Atsushi Kawakami

**Affiliations:** Department of Immunology and Rheumatology, Division of Advanced Preventive Medical Sciences, Nagasaki University Graduate School of Medical Sciences, Nagasaki, Japan.

**Keywords:** Poncet disease, reactive arthritis, spondyloarthritis, tuberculosis

## Abstract

**Rationale:**

Rare cases of reactive arthritis induced by active extra-articular tuberculosis (Poncet disease) have been reported. Complete response to antitubercular treatment and evidence of active extra-articular tuberculosis are the most important clinical features of Poncet disease. We report the case of successfully treated a patient with reactive arthritis induced by active extra-articular tuberculosis with a TNF inhibitor after sufficient antitubercular treatment.

**Patient concerns:**

A 56-year-old Japanese man was admitted to our department with polyarthralgia, low back pain, and high fever. The results of rheumatoid factor, anti-citrullinated protein antibody, human leukocyte antigen B27, and the assays for the detection of infections (with an exception of T-SPOT.TB) were all negative. Fluoro-deoxy-D-glucose–positron emission tomography with CT (PET/CT) showed moderate uptake in the right cervical, right supraclavicular, mediastinal, and abdominal lymph nodes. As magnetic resonance imaging and power Doppler ultrasonography showed peripheral inflammation (tendinitis, tenosynovitis, ligamentitis, and enthesitis in the limbs).

**Diagnosis:**

A diagnosis of tuberculous lymphadenitis was eventually established on the basis of lymph node biopsy results. There was no evidence of a bacterial infection including acid-fast bacteria in his joints, and the symptoms of polyarthralgia and low back pain were improved but not completely resolved with NSAID therapy; in addition, a diagnosis of reactive arthritis induced by active extraarticular tuberculosis was made.

**Interventions:**

The patient experienced persistent peripheral inflammation despite antitubercular treatment for more than nine months and was then successfully treated with a tumor necrosis factor inhibitor (adalimumab 40 mg every 2 weeks).

**Outcomes:**

Finally, the patient responded to the treatment and has been in remission for over 4 months as of this writing.

**Lessons:**

In patients who present with symptoms associated with spondyloarthritis, it is important to distinguish between classic reactive arthritis and reactive arthritis induced by extra-articular tuberculosis infection. Introduction of biological agents should be carefully considered in settings where reactive arthritis induced by active extra-articular tuberculosis shows progression to chronicity despite sufficient antitubercular treatment.

## 1 Introduction

The term “spondyloarthritis” encompasses a number of disorders characterized by axial inflammation (e.g., sacroiliitis and vertebritis) and peripheral inflammation (e.g., arthritis, tenosynovitis, and enthesitis in the limbs). Since the 1970s, it has been recognized that spondyloarthritis has a wider spectrum than previously thought.^[1–4]^ Spondyloarthritis comprises a group of diseases including ankylosing spondylitis, psoriatic arthritis, inflammatory-bowel-disease-related arthritis, reactive arthritis, and undifferentiated spondyloarthritis (an entity that does not fit in any of the other categories).^[3,4]^

In the 1890s, Poncet et al reported the first case of polyarthritis that developed in the presence of active extra-articular tuberculosis with no concomitant evidence of infectious arthritis.^[5]^ Since then, this condition has been referred to as Poncet disease. Subsequently, several cases of Poncet disease have been reported from tuberculosis-endemic regions, especially in the age group of 20 to 40 years.^[6,7]^ Complete response to antitubercular treatment and evidence of active extra-articular tuberculosis are the most important clinical features of Poncet disease.^[8]^

Japan still has a moderate burden of tuberculosis despite being an industrialized country.^[9]^ Elderly people account for a high percentage of Japanese patients with active tuberculosis.^[10,11]^ Aging societies in industrialized countries are more vulnerable to developing tuberculosis.^[10–12]^ Therefore, patients with reactive arthritis induced by active extra-articular tuberculosis may increase even in industrialized countries.

We herein report a patient with reactive arthritis induced by active extra-articular tuberculosis, who experienced persistent peripheral inflammation in the limbs despite antitubercular treatment and was treated successfully with a tumor necrosis factor (TNF) inhibitor.

## 2 Case report

In March 2011, a 49-year-old Japanese man with type 2 diabetes and diabetic nephropathy presented with a high fever and skin rash mimicking erythema nodosum. Although he underwent a detailed examination because of a positive result of T-SPOT.TB, the cause of his symptoms remained unclear. There was no evidence of active tuberculosis, and his symptoms responded to treatment with nonsteroidal anti-inflammatory drugs (NSAIDs). However, in April 2012, he developed pain in the plantar aspect of both feet. Magnetic resonance imaging (MRI) revealed plantar fasciitis, and he responded to low-dose prednisolone (PSL) therapy (5.0 mg/day). In April 2017, he again developed high-grade fever, skin rash mimicking erythema nodosum, and pain in the plantar aspect of both feet at the time of introduction of hemodialysis due to worsening of his diabetic nephropathy. He was successfully treated by restarting low-dose PSL therapy. In September 2017 (age: 56 years), he developed polyarthralgia in the limbs, mechanical low back pain, and a high fever and was subsequently admitted to our department.

At admission, his body temperature was 37.0°C, his blood pressure was 131/54 mmHg, and his heart rate was 71 beats/min. Pulse oximetry revealed 99% oxygen saturation (room air). Physical examination revealed swelling of the left second and fourth fingers and right knee joint. He also had tenderness over the lateral epicondyle of the right elbow, at the right hip joint, around the bilateral knee joints, and over the right plantar fascia. Physical examination of the head, neck, chest, abdomen, skin, and neurological system revealed no abnormalities.

Laboratory investigations showed the following results: white blood cell count, 13,800/μL (neutrophils: 82.0%); hemoglobin, 11.1g/dL; platelet count, 32.1 × 10^4^/μL; C-reactive protein (CRP), 9.54 mg/dL; and erythrocyte sedimentation rate (ESR), 70mm/h. Blood culture showed no evidence of bacterial infection. Although there was an 80-fold increase in antinuclear antibodies, the following immunological and serological results were all negative: rheumatoid factor, anti-citrullinated protein antibody, anti-Ro/SSA antibody, anti-Ro/SSB antibody, anti-double-stranded DNA antibody, anti-Sm antibody, proteinase-3 anti-neutrophil cytoplasmic autoantibodies, myeloperoxidase anti-neutrophil cytoplasmic autoantibodies, and angiotensin-converting enzyme. In addition, the results of the following assays for the detection of infections were all negative (with an exception of T-SPOT.TB): β-D-glucan, human parvovirus B19, hepatitis B and C viruses, human immunodeficiency virus, human T-cell leukemia virus type 1, *Chlamydia trachomatis*, and syphilis. The results of the assays of both cytomegalovirus and Epstein–Barr virus showed a pattern consistent with a past infection. The result of human leukocyte antigen (HLA) B27 was negative.

**Figure 1:**
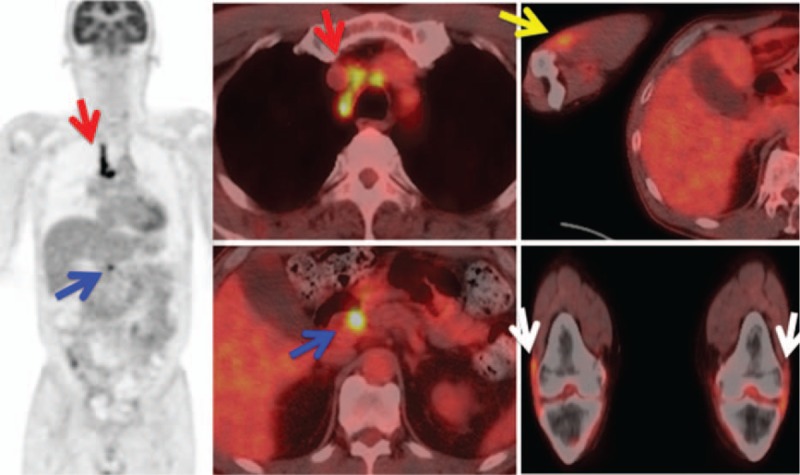
FDG–PET/CT showing moderate uptake by mediastinal (red arrow) and abdominal (blue arrow) lymph nodes, and around the right elbow (yellow arrow) and bilateral knee joints (white arrow).

**Figure 2:**
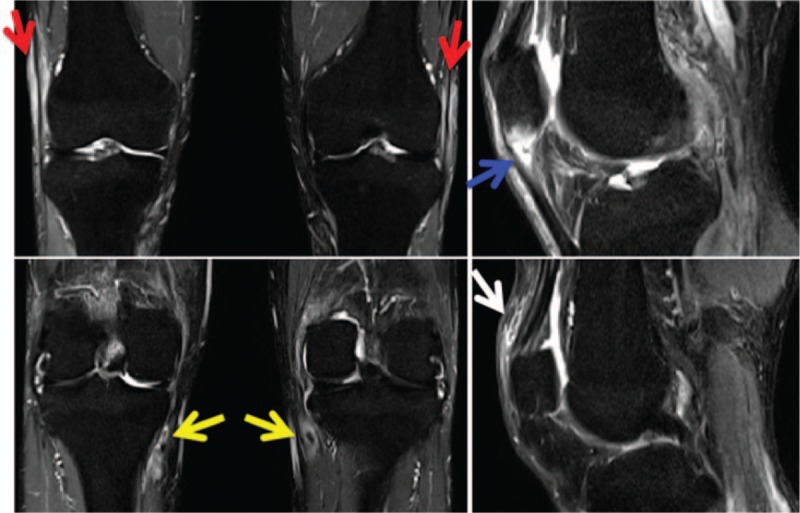
MRI of the bilateral knees, showing ligamentitis of the bilateral iliotibial tract (red arrow), bilateral pes anserine tendinitis (yellow arrow), enthesitis of the right proximal patellar ligament on the inferior pole of the right patella (blue arrow), and enthesitis of the left quadricep tendon on the superior pole of the left patella (white arrow).

**Figure 3:**
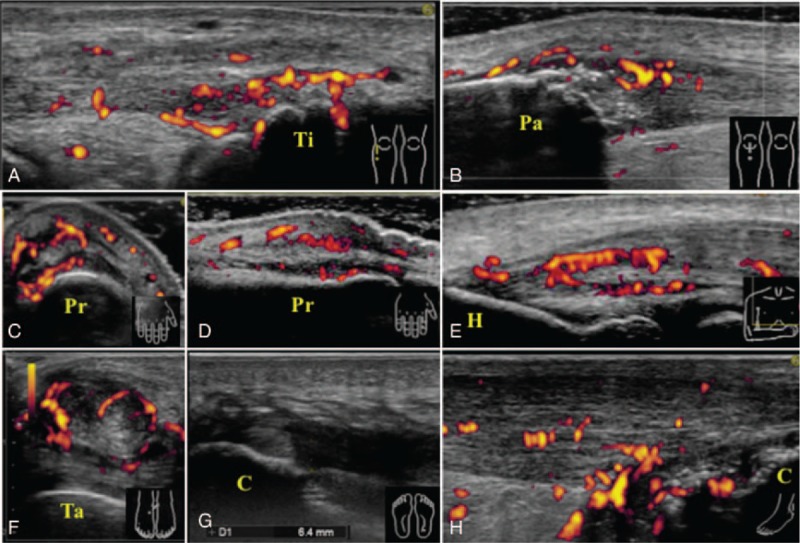
PDUS findings indicating ligamentitis of the bilateral iliotibial tract (A), enthesitis of the right proximal patellar ligament on the inferior pole of the right patella (B), flexor tenosynovitis (C), extensor peritendinitis (D) of the right second finger, common extensor tendinitis at the lateral epicondyle of the right elbow (E), right tibialis posterior tenosynovitis (F), hypertrophy of the bilateral plantar fascia (G), and Achilles tendinitis and enthesitis (H). Ti = tibia; Pa = patella; Pr = proximal phalanges; H = humerus; Ta = talus; C = calcaneus.

Thoracoabdominal computed tomography (CT) revealed no abnormalities except for mild mediastinal lymphadenopathy. Fluoro-deoxy-D-glucose (FDG)–positron emission tomography with CT (PET/CT) showed moderate uptake in the right cervical, right supraclavicular, mediastinal, and abdominal lymph nodes (Fig. 1). In addition, FDG-PET/CT also showed moderate uptake around the bilateral elbow and knee joints (Fig. 1). Although the MRI of the sacroiliac joint and lumbar spine showed no evidence of sacroiliitis or vertebritis, it showed signs of facet arthritis at the left L4/L5 and interspinous ligamentitis. Both MRI and power Doppler ultrasonography (PDUS) of the bilateral knees showed ligamentitis of the bilateral iliotibial tract, bilateral pes anserine tendinitis, enthesitis of the right proximal patellar ligament on the inferior pole of the right patella, and enthesitis of the left quadriceps tendon on the superior pole of the left patella (Figs. 2 and 3A, B). PDUS also showed flexor tenosynovitis and extensor peritendinitis in the right second and fourth fingers (dactylitis), common extensor tendinitis on the lateral epicondyle of the right elbow, right tibialis posterior tenosynovitis, and hypertrophy of the bilateral plantar fascia [Figs. 3C–G].

Based on the results of PET/CT and T-SPOT.TB, we suspected tuberculous lymphadenitis and performed a biopsy of the right cervical lymph node. The biopsy specimen showed epithelioid cell granuloma with Ziehl–Neelsen-positive acid-fast bacteria; therefore, a diagnosis of tuberculous lymphadenitis was established. Culture and polymerase chain reaction (PCR) in synovial fluid of right knee joint showed no evidence of a bacterial infection including acid-fast bacteria. The symptoms of polyarthralgia and low back pain were improved but not completely resolved with NSAID therapy; in addition, the patient was also diagnosed with reactive arthritis induced by active extra-articular tuberculosis (Poncet disease) according to the Amor criteria.^[4]^

**Figure 4:**
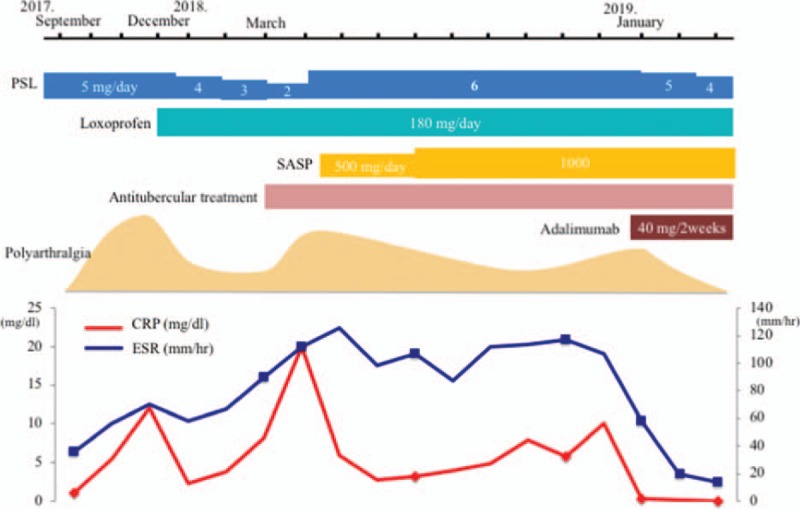
Schematic illustration of the clinical course of the patient. The levels of CRP and ESR and the time course of the therapeutic interventions are shown. CRP = C-reactive protein; ESR = erythrocyte sedimentation rate; PSL = prednisolone; SASP = salazosulfapyridine.

We administered antitubercular treatment with isoniazid, rifampicin, ethambutol, and pyrazinamide and salazosulfapyridine in addition to NSAIDs and low-dose PSL therapy; subsequently, the lymphadenopathy diminished. Although the patient's polyarthralgia and low back pain were relieved after the initiation of treatment, he experienced a relapse of symptoms, which remained persistent. In addition, he developed swelling of the left Achilles tendon; PDUS showed signs of Achilles tendinitis and enthesitis [Fig. 3H]. Finally, after antitubercular treatment for more than nine months, we administered adalimumab 40 mg every 2 weeks. The patient responded to the treatment and has been in remission for over 4 months as of this writing (Fig. 4).

## 3 Discussion

We successfully treated a patient with reactive arthritis induced by active extra-articular tuberculosis with a TNF inhibitor after sufficient antitubercular treatment. To the best of our knowledge, no reports have described detailed imaging findings of multiple ligamentitis or enthesitis induced by active extra-articular tuberculosis and persistence of chronic involvement of joints despite sufficient antitubercular treatment.

Reactive arthritis belongs to a group of diseases known as spondyloarthritis.^[13]^ This disease is characterized by inflammatory back pain, polyarthritis, and extra-articular symptoms (e.g., ocular and mucocutaneous symptoms) following a specific infection.^[14]^ Although the accurate incidence of reactive arthritis remains unclear because of the lack of specific diagnostic criteria,^[15]^ estimates of the annual incidence of reactive arthritis in Europe range from 0.9 to 9.3 per 100,000 population^[16,17]^; the incidence depends on the pathogen frequency in a particular area. Classical reactive arthritis is triggered by gastrointestinal infections (e.g., those caused by *Campylobacter*, *Salmonella*, *Shigella*, or *Yersinia*) or urogenital infections (e.g., those caused by *Chlamydia*); the condition is associated with HLA-B27 positivity and shares typical characteristics of spondyloarthritis, such as axial involvement, tendency for chronicity, and extra-articular manifestations.^[18]^ However, nonclassical reactive arthritis triggered by most other infections typically responds to antibiotic therapy and exhibits no clear association with HLA-B27 positivity; this may contribute to its lower propensity for axial involvement, chronicity, and extra-articular manifestations.^[19]^

Rare cases of reactive arthritis induced by active extra-articular tuberculosis (Poncet disease) or Bacillus Calmette–Guérin therapy for bladder cancer have been reported.^[6,7,20,21]^ The mean age at the onset of Poncet disease was 33.7 ± 12.5 years.^[22]^ Most patients with Poncet disease had active tuberculosis at the site of lymph nodes or active pulmonary tuberculosis.^[8,22]^ Polyarthritis or oligoarthritis is the most commonly reported form of Poncet disease,^[8,22–24]^ whereas tubercular arthritis is typically monoarticular with the involvement of the hip or knee joints.^[25]^ The reported cases of Poncet disease exhibited a predilection for involvement of the lower limb joints; the most commonly affected joints were the ankle, knee, wrist, elbow, and shoulder (in that order).^[8,22]^ When the patient with active extra-articular tuberculosis had polyarticular involvement of especially lower limb joints not monoarticular involvement, we need to suspect reactive arthritis induced by active extra-articular tuberculosis rather than tubercular arthritis.

Poncet disease shares the characteristics of both classical and nonclassical reactive arthritis.^[22]^ Patients with Poncet disease had a higher frequency of HLA-B27 allele as compared to ethnically matched healthy controls,^[26]^ which suggests that genetic susceptibility to spondyloarthritis may also contribute to the onset of Poncet disease after active extra-articular tuberculosis. However, axial involvement and peripheral enthesitis, which are typical characteristics of spondyloarthritis, are considered uncommon among patients with Poncet disease.^[8,27]^ In addition, complete response to antitubercular treatment is one of the major diagnostic criteria for Poncet disease.^[8]^ Some patients with Poncet disease developed arthritis after the initiation of antitubercular treatment, probably due to a form of immune reconstitution. However, all of these patients experienced symptom resolution after the continuation of treatment.^[8,24]^ Although recurrence of joint involvement due to the reactivation of extra-articular tuberculosis has been reported in patients with Poncet disease,^[28]^ chronicity after sufficient antitubercular treatment is unusual.^[8]^

Poncet disease and erythema nodosum caused by active extra-articular tuberculosis may represent different expressions of similar immunopathologic mechanisms.^[7,29]^ Our patient developed recurrent plantar fasciitis following a high fever and skin rash (mimicking erythema nodosum) prior to the onset of polyarthralgia. This finding suggests that he was probably a case of reactive arthritis induced by active extra-articular tuberculosis. However, he had persistent symptoms despite adequate antitubercular treatment. In addition, he showed axial involvement, dactylitis, and remarkable peripheral inflammation (e.g., tenosynovitis, tendinitis, ligamentitis, and enthesitis) rather than arthritis. These findings share more typical characteristics of spondyloarthritis compared to previous reports associated with Poncet disease. This case report illustrates that reactive arthritis induced by active extra-articular tuberculosis may progress to chronicity despite sufficient antitubercular treatment, especially when the patients have more typical characteristics of spondyloarthritis suggesting original predisposition for spondyloarthritis.

In conclusion, we successfully treated our patient having reactive arthritis induced by active extra-articular tuberculosis with a TNF inhibitor after sufficient antitubercular treatment. In patients who present with symptoms associated with spondyloarthritis, it is important to distinguish other types of spondyloarthritis from reactive arthritis, including that caused by active extra-articular tuberculosis. Introduction of biological agents should be carefully considered in settings where reactive arthritis induced by active extra-articular tuberculosis shows progression to chronicity despite sufficient antitubercular treatment.

## Author contributions

**Supervision:** Tomohiro Koga, Momoko Okamoto, Sosuke Tsuji, Ayuko Takatani, Toshimasa Shimizu, Remi Sumiyoshi, Takashi Igawa, Naoki Iwamoto, Kunihiro Ichinose, Mami Tamai, Hideki Nakamura, Tomoki Origuchi, Atsushi Kawakami.

**Writing – original draft:** Yushiro Endo.

**Writing – review & editing:** Shin-ya Kawashiri.
